# ﻿A remarkable new genus of Thripinae (Thysanoptera, Thripidae) without anteocellar setae from India

**DOI:** 10.3897/zookeys.1141.96170

**Published:** 2023-01-19

**Authors:** Remani Rajan Rachana, Bellapu Amarendra, Ramasamy Gandhi Gracy, Katasani Venkata Nagarjuna Reddy

**Affiliations:** 1 National Bureau of Agricultural Insect Resources (ICAR-NBAIR), Bengaluru, Karnataka 560024, India National Bureau of Agricultural Insect Resources Bengaluru India

**Keywords:** Bengaluru, Karnataka, Nandi hills, *Nandithripspouzolziae*, *Pouzolziapetandra* subsp. *wightii*, thrips

## Abstract

*Nandithripspouzolziae***gen. et sp. nov.** (Thripidae, Thripinae) is described from the flowers of Pouzolziapetandrasubsp.wightii (Urticaceae) found in Nandi hills, Karnataka, India. This new genus is characterised by an apomorphy, ocellar setae pairs I and II are both absent, and also has a unique discontinuous pore plate distribution, with a single circular or oval pore plate medially on abdominal sternites II and V–VII of males. Partial mitochondrial cytochrome c oxidase subunit 1 (mtCOI) gene sequence of *N.pouzolziae* was sequenced and the annotated sequence was submitted to NCBI GenBank.

## ﻿Introduction

The Thripinae (Thysanoptera, Thripidae) is the largest of the four subfamilies of the family Thripidae (ThripsWiki 2022). Members of this subfamily are characterised by having the head and legs smooth and without reticulations, the first vein and costa on the fore wing not fused near their base, meso- and metathoracic furca with or without a spinula, and a body that is generally not robustly sclerotised. They exhibit a broad range of plant associations, with many species inhabiting flowers or leaves, some living on both flowers and leaves, many specifically associated with Poaceae, and a few on mosses. A very few species of thrips are predators (Mirab-balou et al. 2013). *Cyrilthripscecidis* Tree & Mound is reported to cause gall induction of plants in Southeast Asia and Australia (Tree and Mound 2009). This subfamily includes almost all thrips species that are considered pests as well as all but one of the vectors of orthotospovirus infections (Mound et al. 2022). Currently, 229 extant genera and 1762 species belonging to this subfamily are known worldwide (ThripsWiki 2022), with 81 genera and 232 species recorded from India (Rachana and Varatharajan 2017).

The objective of this paper is to diagnose a new genus and species from Nandi hills, Karnataka, India. The new species was collected in the flowers of Pouzolziapetandrasubsp.wightii (Benn. & R. Br.) Friis & Wilmot-Dear (Urticaceae), and compare these to related genera.

## ﻿Materials and methods

The specimens were collected by beating leaves and flowers of Pouzolziapetandrasubsp.wightii onto a plastic tray. Specimens were removed with a fine brush into a collecting vial containing 90% ethyl alcohol and mounted onto slides with Canada balsam. They were examined using an Olympus BX 51 microscope and measured using a micrometre eyepiece. Photographs were taken with a Nikon DS-Vi1 camera mounted on a Nikon Eclipse 80i microscope. Keys to genera of the subfamily Thripinae were consulted in diagnosing the new genus (Ananthakrishnan and Sen 1980; Mound and Ng 2009; Masumoto 2010; Mirab-balou et al. 2013). Holotype and paratypes were deposited in the National Insect Museum, National Bureau of Agricultural Insect Resources (ICAR-NBAIR), Bengaluru, India. Using DNeasy Blood and Tissue Kit from Qiagen India Pvt. Ltd. and adhering to the manufacturer's instructions, DNA was extracted from the thrips specimens. The mitochondrial COI gene's standard DNA barcoding region was sequenced for the molecular analysis, and the Universal COI primers (LCO1490/HCO2198) were used in the PCR. Following the manufacturer's recommendations, the amplified products were purified using a Qiagen PCR purification kit, and the purified samples were then sequenced using Sanger's method. Utilizing NCBI Blast tools, the sequence was annotated, and the NCBI GenBank Database was used to generate the accession number.

## ﻿Taxonomic account

### ﻿ Nandithrips gen. nov.

Taxon classificationAnimaliaThysanopteraThripidae

9E4EB9FC-D6A4-540C-A4A5-60199A4D07F3

https://zoobank.org/224FCC78-BFE4-442A-8F15-C38143ED1608

#### Type species.

*Nandithripspouzolziae* sp. nov.

#### Description.

**Female macroptera.** Mouth-cone short and rounded at apex, with 3-segmented maxillary palpi. Ocellar setae pairs I and II absent. Antennae 8-segmented, segment I without median dorsal apical setae, III and IV with forked sensoria, III–VI with a few microtrichial rows (Fig. 5). Pronotum with two pairs of long posteroangular setae, outer pair shorter than inner pair; four pairs of posteromarginal setae, inner pair longer and thicker than the remaining pairs (Fig. 4). Mesonotum with median pair of setae anterior to submedian setae pair. Metanotum with median setae pair at or close to anterior margin, darker and stouter than sub median pair (Fig. 7). Prosternal ferna undivided, narrow at middle; basantra membranous and without setae; prospinasternum broad and transverse. Mesosternal furca with a spinula. Metasternal endofurca without spinula. Fore wing first vein with long gap in setal row, seven basal (first seta transparent) and three distal setae; clavus with five veinal and one discal setae; second vein with 6–9 setae; setae length on both veins increases abruptly beyond distal third of the forewing; posterior fringe cilia wavy (Fig. 8). Tarsi 2-segmented. Hind tibiae and tarsi each with two stout spines at apex. Abdominal tergites without ctenidia but a few microtrichia present on VIII anterolateral to spiracles, tergites without craspedum; tergites VI–VIII with S4 setae minute; tergite VIII with posteromarginal comb, microtrichia absent at middle (Fig. 12); tergite IX with two pairs of campaniform sensilla; tergite X with median slit more than two-thirds (Fig. 9); abdominal sternites without craspedum; sternite II with two pairs of posteromarginal setae, III–VII with three pairs, III–VI with S1, S2, and S3 at posterior margin, VII with S1 and S2 setae placed well ahead of posterior margin, S3 submarginal (Fig. 13). Sternites without discal setae. Ovipositor well developed.

**Male macroptera.** Abdominal tergite IX without median short and stout setae (Fig. 10); sternites II and V–VII each with a circular or oval pore plate medially (Fig. 11).

#### Etymology.

In reference to the type locality.

#### Generic relationships.

The absence of ctenidia on the abdominal tergites indicates that *Nandithrips* is not related to either the *Thrips* or *Frankliniella* genus groups (Mound and Palmer 1981). However, *Nandithrips* shares the apomorphic character, the lack of ocellar setae pair II, only with the African genus, *Bournierothrips* Bhatti, which is a member of the *Thrips* genus group. This character state appears to be a convergence, as this genus does not belong to the same genus group. *Bournierothrips* has ctenidia and other character states of the *Thrips* genus group and the lack of the ocellar setae pair II seems to be an additional loss in that lineage which already lacks ocellar setae pair I. Even though both the genera share a unique apomorphic character within the subfamily Thripinae, they may not be closely related. The host plant association of the two genera appears to be different: this genus was collected in the flowers of Pouzolziapetandrasubsp.wightii, but all described *Bournierothrips* species are associated with mosses, and the genus is endemic to Africa.

The lack of microtrichial fields laterally on the abdominal tergites indicates that this genus is not related to *Scirtothrips* genus-group (Masumoto and Okajima 2007), and presence of long setae on the pronotum suggests that it is not related to *Anaphothrips* genus group (Mound and Masumoto 2009). The general appearance of *Nandithrips* suggests that it is not related to *Taeniothrips* genus group even though it shares some character states like the absence of ocellar setae I and ctenidia (Mound and Palmer 1981; Wang et al. 2020). The absence of a pair of dorsoapical setae on the first antennal segment indicates that it is not related to the two major genus-groups centred on *Trichromothrips* and *Mycterothrips* (Masumoto and Okajima 2005, 2006), even though *Nandithrips* shares several characters with *Trichromothrips* genus group like the absence of ocellar setae pair I, ctenidia, craspeda, and discal setae on sternites and the position of S1 and S2 setae on sternite VII.

It is similar to the Old World flower-inhabiting genus, *Lefroyothrips* Priesner in colour, appearance, the absence of paired dorso-apical setae on antennal segment I, sculpture and chaetotaxy of the meso- and metanota, the absence of ctenidia and craspeda, and the presence of a group of microtrichia anterior to spiracle on abdominal segment VIII; however, *Nandithrips* is distinguished from *Lefroyothrips* in lacking ocellar setae pair I, the tergite VIII with the posteromarginal comb interrupted medially, the position of S2 setae on abdominal sternite VII, the pore gland shape and distribution on the sternites of males, and the stout thorn-like setae on tergite IX of males absent. Many of the characters of *Nandithrips*, particularly the absence of a pair of dorso apical setae on the first antennal segment, are shared with species of the flower-inhabiting genera *Ceratothrips* Reuter and *Projectothrips* Moulton. However, *Nandithrips* differs from *Ceratothrips* by lacking ocellar setae pair I, tergite VIII with the posteromarginal comb interrupted medially, the position of S1 and S2 setae on abdominal sternite VII, and the pore gland shape and distribution on the sternites of males. *Projectothrips* is a highly distinctive genus because of the elongate, slender, eighth antennal segment that is about nine times as long as wide. This genus shares several character states with the members of *Megalurothrips* genus group (*Craspedothrips* zur Strassen, *Megalurothrips* Bagnall, *Odontothripiella* Bagnall, and *Odontothrips* Amyot & Serville) and *Ceratothripoides* Bagnall, *Retanathrips* Mound & Nickle, and *Pezothrips* Karny. However, the absence of a pair of dorsoapical setae on the first antennal segment indicates that it is not related to these genera. Even though Mound and Palmer (1981) included *Ceratothripoides*, *Ceratothrips*, *Craspedothrips*, *Lefroyothrips*, *Megalurothrips*, *Odontothripiella*, *Odontothrips*, and *Projectothrips* in the *Megalurothrips* genus group, *Ceratothrips*, *Lefroyothrips*, and *Projectothrips* may not belong in this group because of the absence of dorso-apical setae on antennal segment I (Masumoto and Okajima 2020). Moreover, Zhang et al. (2019) showed in their phylogenetic analysis based on morphological data that *Craspedothrips*, *Megalurothrips*, and *Odontothrips*, genera with dorsoapical setae on antennal segment I, are included in the same clade and this clade was the sister-group to *Mycterothrips* Trybom, not *Ceratothripoides*. According to their analysis, *Ceratothripoides* seems to be the sister group of *Pezothrips*, but the systematic positions of these two genera are unresolved.

Mound and Palmer (1981) indicated that the absence of ocellar setae I is an apomorphic condition and presence/absence of this setae pair appears to be remarkably constant within genera and genus groups within the subfamily Thripinae. They also mentioned that ocellar setae pair II is remarkably constant in size and position. Hence, after examining multiple specimens (59 females, 22 males) of this genus, we assume that this apomorphic character, the absence of ocellar setae II, is constant within *Nandithrips*. Minaei and Mound (2021) stated that character-state reversals have often been interpreted as apomorphies, such that an unusual looking species is given separate taxonomic status on the basis of the absence of a single character state and, moreover, loss of a character occurs quite commonly. They also stressed the importance of evaluating a new taxon in relation to the structure of closely related taxa under circumstances of apparent absence or loss of a character state. Understanding well the depth of their observations, and after examining multiple specimens of both sexes, we ascertain that the absence of ocellar setae II is stable across all the examined specimens and looked for the other characters which justify its diagnosis as a new genus. One more character state which is unique to *Nandithrips* is the pore plate distribution in males, and this character is very important in discussing the novelty of taxa if males are known. In the subfamily Thripinae, wherever males are known, eight groups of pore plate distribution has been suggested: medially on sternites III + IV + V (± VI, VII, and VIII); medially only on sternites indicated (III, III–IV, and VII); C-shaped pore plate on sternites III + IV + V (± VI and VII); two or three pore plates on several sternites; multiple small pore plates on at least III–VI; on antecostal ridge of at least IV–VI (rarely only II); gland aperture on antecostal ridge of III (no pore plate), and pore plates or glandular structures absent (Mound 2009). However, *Nandithrips* has a unique discontinuous pore plate distribution with a single circular or oval pore plate medially on sternites II and V–VII, and this condition is not shared with any of the genera in the subfamily Thripinae, wherever males are known. In the new genus, an abrupt increase in setae length on both the veins beyond the distal third of the fore wing is noticeable, which is also not shared with any other genera in the subfamily Thripinae.

To conclude, although *Nandithrips* is a member of the subfamily Thripinae, more precise relationships are not clear.

### ﻿ Nandithrips pouzolziae sp. nov.

Taxon classificationAnimaliaThysanopteraThripidae

C02D3B23-EDAF-5073-9C6B-D018E37F3914

https://zoobank.org/D2708492-9BDC-479A-885E-A39BE4F1E9F6

#### Type material.

**Figure 1.**
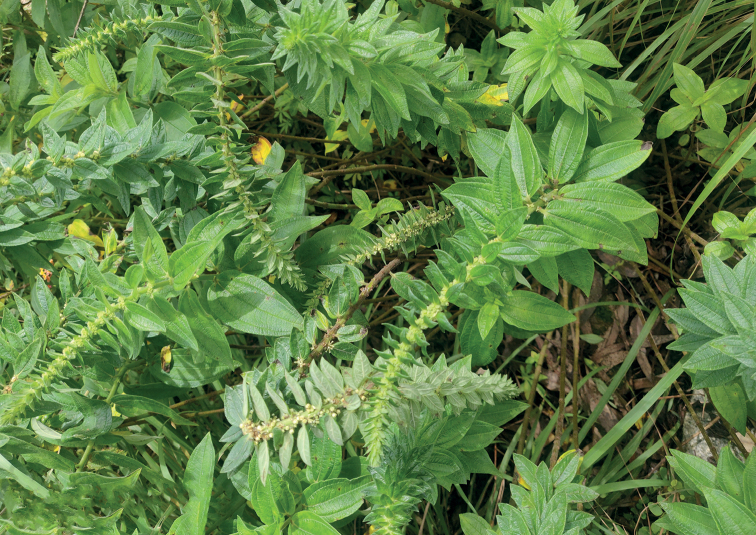
Pouzolziapetandrasubsp.wightii.

***Holotype*** female, Nandi hills (13.37°N, 77.68°E), Bengaluru, Karnataka, India, in the flowers of Pouzolziapetandrasubsp.wightii (Fig. 1), 16 September 2022, Amarendra B. (ICAR/NBAIR/THYS/16092022). ***Paratypes*** 58 females, 22 males with same data as holotype.

#### Description.

**Figures 2–8.**
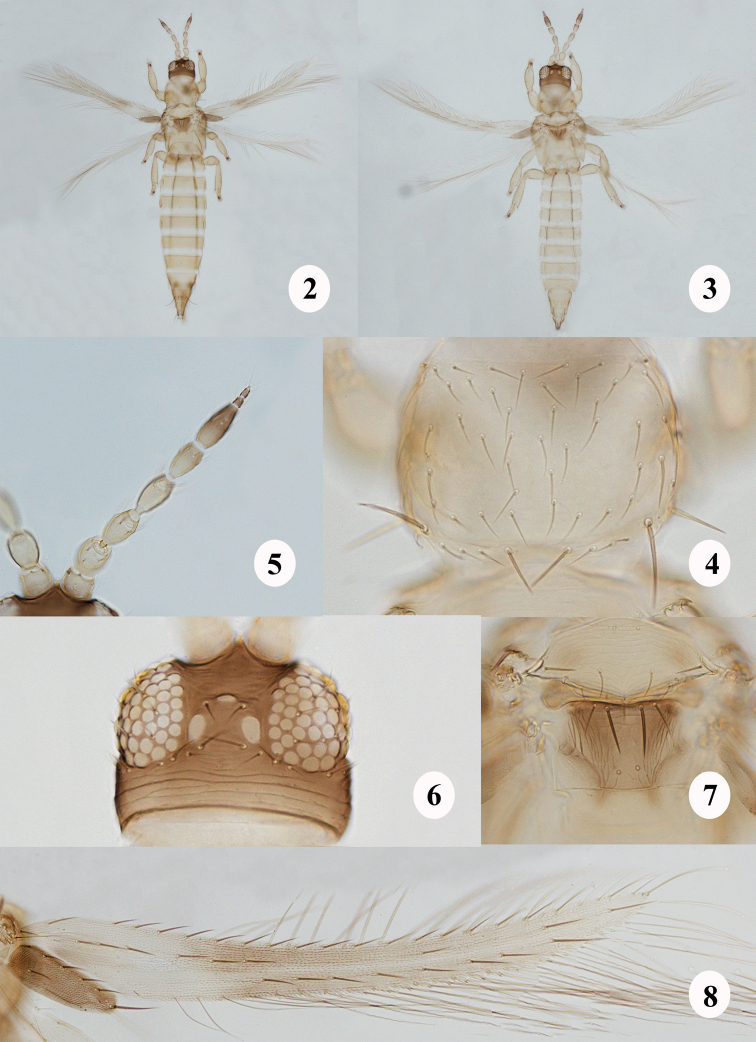
*Nandithripspouzolziae* sp. nov. **2** female **3** male **4** prothorax **5** antenna **6** head **7** pterothorax **8** fore wing.

**Female macroptera (Fig. 2).** With the character states given in the generic diagnosis above. Body golden-yellow except head, metanotum and clavus brown; antennal segments I–IV pale yellow, V yellow basally and shaded brown apically, VI brown with base slightly pale, VII and VIII brown (Fig. 5); fore wing slightly dusky except pale base and apex (Fig. 8); all legs yellow; prominent body setae pale to brown. Head wider than long; ocellar setae pair III situated at the tangent between the fore and hind ocelli. Postocular setae six pairs, pairs I and III subequal and the longest, pair V situated far from pair IV (Fig. 6). Antennal segment II without microtrichial rows, III–VI with microtrichial rows, III–IV with apical neck, III–V with pedicel (Fig. 5). Pronotum weakly sculptured with transverse striae (Fig. 4). Mesonotum sculptured with transverse anastomosing striae; campaniform sensilla present anteromedially. Metanotum with irregular transverse lines anteriorly, irregular reticulate sculpture medially, longitudinal striations laterally; campaniform sensilla present (Fig. 7). Abdominal tergite I transversely striate; II–VIII with a few striations laterally. Abdominal sternites without discal setae.

Measurements (holotype female in microns). Body length 1200. Head, length 90; width across eyes 115; ocellar setae III 18; postocular setae I 23. Pronotum length 100; width 143; outer posteroangular setae 38; inner posteroangular setae 58. Fore wing length 520. Antennal segments III–VIII length 40, 35, 33, 38, 5, 8.

**Male macroptera (Fig. 3).** General structure as in female but smaller. Abdominal tergite IX with S1 and S2 setae subequal in length, S2 setae positioned anterior to S1 setae (Fig. 10); sternites II and V–VII each with a circular or oval pore plate medially (Fig. 11).

Measurements (paratype male in microns). Body length 850. Head, length 70; width across eyes 100; ocellar setae III 13. Pronotum, length 88; width 125; outer posteroangular setae 33; inner posteroangular setae 40. Fore wing length 450. Antennal segments III–VIII length 38, 35, 28, 35, 5, 8.

#### Etymology.

In reference to the host plant of this species.

#### Molecular characterization.

A partial mtCOI gene of *N.pouzolziae* was sequenced and the annotated gene sequence was deposited in the National Centre for Biotechnology Information (NCBI) database, accession number OP714094.

**Figures 9–13.**
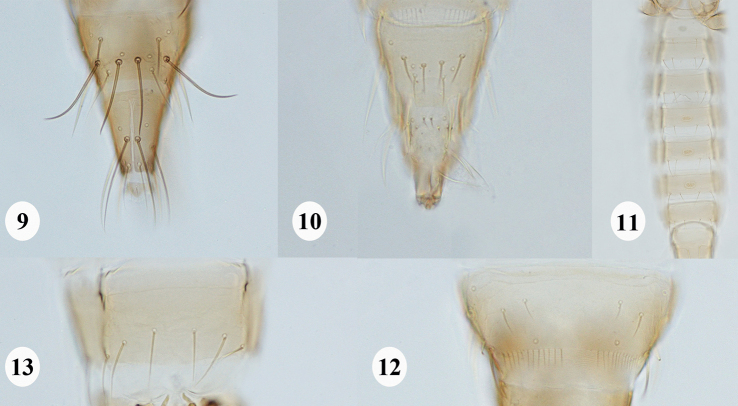
*Nandithripspouzolziae* sp. nov. **9** female abdominal tergites IX–X **10** male abdominal tergites IX–X **11** pore plate on abdominal sternites II and V–VII **12** abdominal tergite VIII **13** abdominal sternite VII.

## Supplementary Material

XML Treatment for Nandithrips

XML Treatment for Nandithrips pouzolziae
